# Lessons from the frontline: The value of emergency care processes and data to pandemic responses across the Pacific region

**DOI:** 10.1016/j.lanwpc.2022.100515

**Published:** 2022-07-06

**Authors:** Rob Mitchell, Gerard O'Reilly, Lisa-Maree Herron, Georgina Phillips, Deepak Sharma, Claire E. Brolan, Sarah Körver, Mangu Kendino, Penisimani Poloniati, Berlin Kafoa, Megan Cox

**Affiliations:** a School of Public Health and Preventive Medicine, Monash University, Melbourne, Australia; b Emergency & Trauma Centre, Alfred Hospital, Melbourne, Australia; c School of Public Health, Faculty of Medicine, The University of Queensland, Brisbane, Australia; d Emergency Department, St Vincent's Hospital Melbourne, Melbourne, Australia; e Emergency Department, Colonial War Memorial Hospital, Suva, Fiji; f Centre for Policy Futures, Faculty of Humanities and Social Sciences, The University of Queensland, Brisbane, Australia; g Emergency Department, Port Moresby General Hospital, Port Moresby, Papua New Guinea; h Emergency Department, Vaiola Hospital, Nuku'alofa, Tonga; i Public Health Division, Secretariat of the Pacific Community, Suva, Fiji; j Faculty of Medicine and Health, The University of Sydney; NSW, Australia; k The Sutherland Hospital, NSW, Australia; l NSW Ambulance, Sydney, Australia

**Keywords:** COVID-19, Emergency care, Emergency medicine, Health systems, Health system building blocks, Health data, Pacific Islands, Pacific region

## Abstract

**Background:**

Emergency care (EC) addresses the needs of patients with acute illness and injury, and has fulfilled a critical function during the COVID-19 pandemic. ‘Processes’ (e.g. triage) and ‘data’ (e.g. surveillance) have been nominated as essential building blocks for EC systems. This qualitative research sought to explore the impact of the pandemic on EC clinicians across the Pacific region, including the contribution of EC building blocks to effective responses.

**Methods:**

The study was conducted in three phases, with data obtained from online support forums, key informant interviews and focus group discussions. There were 116 participants from more than 14 Pacific Island Countries and Territories. A phenomenological approach was adopted, incorporating inductive and deductive methods. The deductive thematic analysis utilised previously identified building blocks for Pacific EC. This paper summarises findings for the building blocks of ‘processes’ and ‘data’.

**Findings:**

Establishing triage and screening capacity, aimed at assessing urgency and transmission risk respectively, were priorities for EC clinicians. Enablers included support from senior hospital leaders, previous disaster experience and consistent guidelines. The introduction of efficient patient flow processes, such as streaming, proved valuable to emergency departments, and checklists and simulation were useful implementation strategies. Some response measures impacted negatively on non-COVID patients, and proactive approaches were required to maintain ‘business as usual’. The pandemic also highlighted the value of surveillance and performance data.

**Interpretation:**

Developing effective processes for triage, screening and streaming, among other areas, was critical to an effective EC response. Beyond the pandemic, strengthening processes and data management capacity will build resilience in EC systems.

**Funding:**

Phases 1 and 2A of this study were part of an Epidemic Ethics/World Health Organization (WHO) initiative, supported by Foreign, Commonwealth and Development Office/Wellcome Grant 214711/Z/18/Z. Co-funding for this research was received from the Australasian College for Emergency Medicine Foundation via an International Development Fund Grant.

**Research in context**

**Evidence before this study**

‘Processes’ and ‘data’ have been nominated as essential building blocks for Pacific emergency care (EC) systems. The relevance of these areas to EC delivery during the pandemic has not been explored, especially in the context of the Pacific region.

**Added value of this study**

This prospective, qualitative study investigated the experiences of Pacific EC clinicians responding to the pandemic. It has established the specific processes and aspects of data management that have been of most concern to EC stakeholders in Pacific Island Countries and Territories. In particular, it has unpacked the enablers and barriers of various EC process measures, such as triage, screening, streaming (zoning) and patient flow. In doing so, the value of checklists, simulation and systematised data collection has been highlighted.

**Implications of all the available evidence**

The study has established that ‘processes’ and ‘data’ have been central to the delivery of safe and timely EC during the pandemic, just as they are under routine conditions. Enhancing the capacity of EC clinicians to implement effective process and data management solutions will result in more resilient EC systems.

Alt-text: Unlabelled box

## Introduction

Emergency care (EC) is an essential component of high-performing and agile health systems. It addresses the needs of patients with acute illness and injury, and fulfils a critical function during surge events and disasters.^1^^,^^2^ EC is also a tool for achieving universal health coverage, and has the potential to address a significant burden of morbidity and mortality in low- and middle-income countries (LMICs).^1^, ^2^, ^3^, ^4^ As with many other disease processes,^5^^,^^6^ COVID-19 has highlighted how timely medical care can improve health outcomes at both patient and population levels.^7^, ^8^, ^9^, ^10^, ^11^, ^12^, ^13^, ^14^

**Figure 1:**
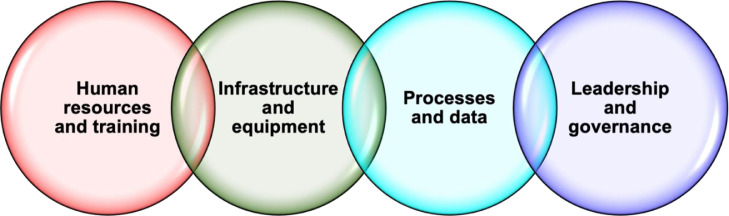
World Health Organization health system building blocks, adapted for the Pacific emergency care context and this qualitative research project.^15^^,^^17^

EC is a systems-based discipline, integrating community, pre-hospital and facility-based care.^1^^,^^2^ It follows that effective processes are central to ensuring patients receive the right care at the right time and the right place. In the Pacific region, ‘processes’ has been nominated as one of the building blocks of an effective EC system, and a priority area for EC development (Figure 1).^15^ Similarly, the essential role of EC ‘data’ in defining disease burden and outcomes, measuring access and resource utilisation, monitoring performance, assisting with public health surveillance and informing evidence-based health planning has been recognised.^15^^,^^16^

By global standards, the Pacific region has been relatively successful at containing the spread of the virus among unvaccinated populations.^18^ The relative isolation of Pacific Island Countries and Territories (PICTs), combined with significant border restrictions, has served to minimise transmission and mitigate the impact on health services.^18^ Where large-scale outbreaks have occurred, in Fiji and Papua New Guinea for example, EC clinicians and systems have been central to both clinical and public health responses.^17^, ^19^, ^20^, ^21^, ^22^ As in other global regions, the development and deployment of effective EC processes has been critical. Examples highlighted by previous research include referral pathways that connect community and hospital-based care; emergency department (ED) triage; and patient flow strategies that aim to minimise ED crowding.^17^, ^20^, ^21^, ^22^, ^23^

This paper reports selected findings from a prospective, qualitative research project that aimed to explore the impact of the pandemic on EC clinicians and other key stakeholders across the Pacific region. It is focussed on the EC building blocks of ‘processes’ and ‘data’. The research context and broader findings have been summarised elsewhere.^17^^,^^24^

## Methods

This study was conducted as a collaboration between Australian and PICT researchers, and employed prospective, qualitative research methods grounded in a phenomenological methodological approach.^25^^,^^26^ Study methods have been described in detail previously.^17^

We gathered data from PICT EC clinicians and other relevant stakeholders between March 2020 and July 2021 (Table 1). Informed consent was obtained from research participants. Phases 2 and 3 utilised semi-structured interview and discussion guides developed by the research team.^17^

**Table 1:** Data collection phases and study participants.

**Phase 1**	Online support forums via Zoom^27^, ^28^	• There were 13 online support forums hosted by SPC and ACEM between March and October 2020^27^ • More than 80 active participants (EC clinicians and stakeholders) from PICTs (and some non-Pacific countries) voluntarily engaged in the online discussion
**Phase 2**	In-depth interviews via Zoom^28^^,^^29^	• Semi-structured interviews of 45–90 min duration were conducted with 13 key informants in Fiji, Kiribati, Palau, Papua New Guinea, Samoa, Solomon Islands, Timor Leste, Tonga and Vanuatu • These key informants were purposively selected because they had coordinated EC in a PICT during the COVID-19 pandemic
**Phase 3**	Focus group discussions via Zoom^28^^,^^29^	• Three focus groups were conducted with EC stakeholders from the recognised Pacific regions of Micronesia (Federated States of Micronesia, Kiribati, Marshall Islands, Nauru, Palau and the northern Pacific states), Polynesia (Cook Islands, Samoa, Tokelau, Tonga, Tuvalu and other Small Island states) and Melanesia (Fiji, Papua New Guinea, Solomon Islands and Vanuatu, as well as Timor Leste)^30^

Data collected during each phase were digitally recorded with participant permission, transcribed verbatim, and subsequently de-identified to protect participants’ anonymity. All data underwent preliminary coding using QSR NVivo^31^ and a hybrid inductive (data driven)^32^ and deductive approach.^33^ Deductive codes were derived from the World Health Organization health system building blocks, as adapted for the Pacific EC context (Figure 1).^15^

Data related to ‘processes’ and ‘data’ were thematically analysed by RM and GOR. Emerging themes and tentative findings were presented to the broader research team to facilitate verification through discussion and data triangulation. Thematic findings were further analysed to identify enablers of, and barriers to, effective EC responses.

Ethics approval and registration was provided by The University of Sydney Human Research Ethics Committee (Reference 2020/480) and Monash University Human Research Ethics Committee (Reference 28325) respectively. Research protocols for Phases 1 and 2A of the research were also reviewed by the World Health Organization's AdHoc COVID-19 Research Ethics Review Committee (Protocol ID CERC.0077) and declared exempt. Reporting of study data adheres to Enhancing the Quality and Transparency of Health Research (EQUATOR) and Standards for Reporting Qualitative Research (SRQR) guidelines.^34^^,^^35^ Funders had no role in study design, results analysis or manuscript preparation.

## Results

In total, 116 participants from more than 14 PICTs participated in the study. Five key themes emerged from the analysis of informant responses on ‘processes and data’:

1. Implementing effective triage and screening.
2. Instigating streaming (zoning) and optimising patient flow.
3. Developing standard operating procedures and checklists.
4. Maintaining ‘business as usual’.
5. Enhancing surveillance and data reporting.

Sub-themes, delineated as enablers (strengths) and barriers (gaps), are presented in Table 2. The table includes indicative quotes from study informants.

**Table 2:** Enablers and barriers related to key themes, with exemplar quotes from study informants.

Enablers	Barriers
**1. Implementing triage and screening**	
Access to clear and consistent guidance, including triage and screening criteria *“We're fortunate to have the guidelines of WHO and CDC, prior to [the arrival of] COVID”*	Lack of pre-existing processes and local experience with disaster response *“We don't in our training have much exposure to disaster management and so [] it was quite challenging to lead the COVID response and to get the systems up and running. [] I had to micro-manage everything: setting up the triage, the pre-triage… Having to micro-manage everything was probably the most difficult thing for me in the COVID response in [our hospital]”*
Effective referral and communication pathways between healthcare facilities *“Before they send or refer a case whoever is referring, [we'll] update the ED first, ‘Look we're sending this case over to you guys and this is what we [referral hospital] have done’… We're hoping with the establishment of the ambulance and the emergency centre in [our country] [they can] help us solve the burden of work especially in terms of choosing which patients [are or aren't] supposed to be referred [] to [the] ED”*	Sub-optimal referral and communication pathways between healthcare facilities *“The biggest barrier I would say is the communications…. For instance, like now, having a simple patient flow from the border district to come to [the] COVID centre is still a problem”*
Staff enthusiasm and responsiveness *“Here,* [setting] *up the COVID triage quickly* [] *wasn't perfect when we were given the building. I must say thank you very much to my nurses who did the bulk of the job in ensuring that the flow was set up and that things were clearly labelled”*	Lack of support from hospital leaders *“Our executive doesn't really understand us, in what we're trying to do to keep our patients and staff safe. We're trying to create this SCOVID screening and yet our management, the hospital executive, didn't actually put in an effort to address it for us. We're trying at our end and the other end is not working. We're hitting against a brick wall at this point in time. We really want to set up something that will keep us safe with our patients, but [the protection] still hasn't come”*
**2. Instigating streaming and optimising patient flow**	
Recognition of the value of efficient EC processes from previous disease outbreak experience *“Given our increased role with other outbreaks, like leptospirosis and measles, [and] when COVID came because we were one of the hospital entry points for all our hospital facilities; we were the frontliners of the [hospital] frontliners. Our input was more taken into consideration because it made a difference to the rest of the hospital who we allowed in and who we didn't…. So very early on… they realised that the plans and processes that we're [already]adopting to be more COVID safe and very useful. So there was more of that recognition and realisation of the importance that made other areas in the hospital speak [to] the same tune and use the same processes”*	Lack of pre-existing patient flow processes *“Before COVID we didn't have any sort of patient flow system if you work here; it's like any Tom, Dick and Harry will just come in”*
Contributions of non-clinical staff *“I talked with the cleaners and the porter. I emphasised to them how important it is - cleanliness in the department and also at home, to ensure we don't bring any infection from the hospital there and vice versa. And the importance of ensuring that the alcohol bottles are always filled, and there's always soap at the sink”*	Cumbersome communication pathways with inpatient teams *“The idea was that this was going to be a one-call system, where once the patient needs admission they go straight to the ward, we just call one number. But unfortunately at present our registrars are still having to call three, four people”*
**3. Developing standard operating procedures and checklists**	
Simulation and exercises “*The day before they conducted a mock exercise where they were bringing patients, actors, to the rural clinic, where they were identified, brought to the hospital, triaged, and sent to the isolation ward. Just to check how we're going and identify any problems, which there were quite a few. But it was our first run, so it wasn't entirely unexpected. Mainly just procedures, and communication problems. So not, unachievable”*	Limited supply of clinician leaders *“It was a lot of just everyday going through the systems, trying to reinforce how systems should change in the context of COVID. Everything from how linen was taken care of, how garbage was managed, how patients flowed through the hospital, how specimens were handled and that kind of thing. It was difficult to just be the one person to reinforce the systems and adapt them to COVID”*
Access to up-to-date epidemiological information to inform process changes and address uncertainty *“[At first] everybody was very worried and concerned; the fear of the unknown. But then very early on, given that we had people within those [national emergency operations] committees [] we were able to get real-time information [and] were able to feedback correct, appropriate information to the staff. And they were very appreciative of it. So that fear and sense of panic was not there; there was more of a sense of ‘Okay, I know that if I'm unsure I can ask these people and they will give me the right information’, and not, like we say, our ‘coconut wireless’ information”*	Inconsistent guidelines and policies *“…Someone has their own guideline on who's low and who's high risk. So there's* [] *confusion among staff. I must say* [although] *it's going well, the important thing is that communication and information must be laid across so everybody* [has the same conceptual understanding]”
**4. Maintaining ‘business as usual’**	
Previous experience in disaster, outbreak and surge event response *“In the past [we've] had a bit of preparation for SARS. So without any science out there, we grew our plans for COVID-19 out of [our] SARS activity”*	Closure of routine clinics and outpatient services “*We've neglected a lot of non-COVID issues. We need to really get on that quick… we've gone back a couple of years of all the good work that has been done with what has happened … We've gone back about five years. Cancer, cancer cases – on a good day we already had a big list, people wait for so long to try and get operated on. Now there's no operation, what's happening? … For the Pacific yet, we haven't seen the impacts of COVID but there's definitely a lot more impact on non-COVID cases I must say”*
Staff motivation to improve healthcare quality and safety *“We want to see improvement of our system. That would mean infrastructure, the staff, the whole of the system. We want it to be improved from what we currently face now. Making all the SOPs relevant to* [our country]*. We don't want the copy and paste from somewhere else, we want something that is relevant in our country. We want it to be locally made so that it suits us in every way.”*	Suspension of normal referral and escalation pathways *“One thing that we would like to do away with, that is currently a reality, is the fact that we can't refer cases. Treatable, surgical cases that would otherwise have a chance of improving [a person's] quality of life or even just their survival. We've lost an opportunity”*
Vision and commitment from emerging EC leaders *“I saw this department [ED] as the worst department within the hospital. I think it's the same everywhere else. But whatever small contribution I can give [is] my driving factor - to get this department into a better state than when I started off. I don't have the qualifications to try and do that, but whatever small contribution I can do with a small young team, I think we can build on this []. I think COVID just came in as a bonus [] to learning how to better prepare us for the long run”*	Competing interests for care “*And since the start of the pandemic we were also having an outbreak of dengue as well. Two years in a row we were dealing with a type 3 and type 4 dengue, so we were still coming out of that outbreak when we met the pandemic of COVID-19. We found ourselves very unprepared for our response.”*
Collaboration with other medical disciplines “*Luckily we had our taskforce, so we had the medical consultant, the ED, surgeon, paediatrician and everybody at one place, so they quickly devised what we'd do with a patient who presented at any place and time.”*	Lack of primary care capacity “*The real challenge for us* [is] *we have very weak system*[s] *– primary health is pretty much non-existent. We're trying to tighten up that area”*
**5. Enhancing surveillance and data reporting**	
Pre-existing syndromic surveillance processes *“And of course…. [we want] to improve from the current surveillance systems that we have in place. So currently we have the syndromic surveillance for the Pacific; we do have the syndromic surveillance for [our country]. In the future we would like to include COVID [symptoms] inside the syndromics, so that whatever we pick during our surveillance can help us in our future planning”*	Lack of systematised data collection and reporting in the ED “*There was not much data…basically I started from zero”*

### Theme 1: Implementing effective triage and screening

Participants reflected extensively on the pivotal role of triage and screening in ensuring patient and staff safety, especially in ED settings. These processes were widely regarded as essential to an effective EC response.

Triage and screening serve discrete but related functions. The aim of triage is to categorise the urgency of a patient's condition, ensuring that those with time-sensitive medical needs are prioritised. Screening, meanwhile, is focused on the determination of transmission risk, with the objective of identifying individuals with confirmed or suspected COVID-19 (SCOVID) (ie, patients with symptoms of, or epidemiological risk factors for, SARS-CoV-2 infection). This process allows the separation of high transmission risk patients (ie, those with confirmed or suspected COVID-19) from others.

In some PICT settings, these triage and screening functions were integrated and performed at a single ‘checkpoint’ on entry to the ED or healthcare facility. Several participants described how these processes worked at their hospitals. For example:

*“…We [] sort those who are presenting with [or without] respiratory symptoms []. Those with no respiratory symptoms will be directed straight to ED but those with respiratory symptoms, we will actually use two screening methods [including] the case definition from WHO. If they do feel ill [they become SCOVID patients and they'll]... be taken away immediately to a designated area”*

The value of the screening process was highlighted when EDs experienced inadvertent exposure events, leading to the infection of patients and/or staff:

*“So after that incident...we decided to, [in] our triage area, to make sure that before patients come in, they actually are screened for respiratory [symptoms]....”*

In many settings, the outcome of the screening assessment was used to stream the patient (ie, define the location of care). As highlighted below, patients who ‘screened positive’ could then be cared for in a dedicated ‘respiratory zone’ or isolated treatment space within the ED:

“*With COVID we developed certain processes where we now have screening before triage, and we had to have our designated isolation room with the emergency department, which is a bit separate from our open area where we would see our normal business patients”*

In many instances, ED staff were called upon to facilitate screening for the hospital as a whole, and not just patients with EC needs. This placed considerable strain on limited human resources for EC:

*“We were asked to…set up and to man a COVID triage, which is in a separate building … on the other side of the hospital. We were asked to look after the COVID triage, in case a sick COVID patient [came] through. And also, at the same time, run the ED”*

Settings with pre-existing triage systems were better placed to scale up their capacity for both triage and screening, and ensure consistent use of criteria across hospital and community settings. In some contexts, ED clinicians played a pivotal role in training community healthcare workers in these processes. For instance, in one PICT:

“*In the facilities [staff were] trained on triage, sorting and receiving patients and the basic fever triage. All facilities in [our city] started fever triage which also helped their assessment for acuity of patients and smoothed ambulance referrals.”*

Participants agreed that a lack of experience with triage, and inconsistent guidelines for screening, created uncertainty among clinicians. While this generated fear in some instances, it also accelerated the implementation of new processes:

*“Lack of information just caused a lot of fear…there was no clear guidelines in place. Some people were worried about staffing - where do we get additional people to come and go and look after our systems? I know for emergency care they were all worried about triaging, and how do you do that…”*

Participants discussed how PICT EDs employed a variety of strategies to support these new processes, including the establishment of ‘forward’ triage and the use of simple measures to delineate checkpoints, treatment streams and care zones:

*“We have since stuck down red tape [on the ground] next to the ambulance entrance showing where they have to stop outside and wait. Low tech but [it] seems to make people stop!”*

In some settings, EC clinicians were deeply frustrated by a lack of support from hospital leaders in relation to the establishment of effective triage and screening. This was particularly the case for settings with low case numbers:

*“The biggest challenge is trying to set up the SCOVID screening area in front of the ED. It hasn't happened. We tried to meet with the executive, our leaders, but still now we're unable to establish it…At the moment we don't have any case[s], and we're still trying to set up the ED SCOVID screening area”*

In establishing triage and screening, PICT clinicians drew on their experience from previous surge events and disasters. Study participants described how lessons learnt from other communicable disease outbreaks informed their approaches, even in the absence of local transmission of COVID-19.

### Theme 2: instigating streaming (zoning) and optimising patient flow

Participants described how PICT EC clinicians rapidly identified the value of ‘zoning’ their departments in order to separate high transmission risk patients from others. In many cases, this required process reform to ‘stream’ patients into designated high-risk areas, including the development of testing protocols as well as ‘clearance’ criteria for patients with suspected COVID-19. Several informants reflected on how they adapted their models of care for this purpose:

“*In terms of our ED…we've divided the teams into respiratory and non-respiratory ED, which will start from hopefully tomorrow or the day after. There's a clear demarcation so the current ED… will serve as our COVID ED or respiratory ED”*

Participants acknowledged, however, that in many instances this division had to be achieved with sub-optimal infrastructure and limited resources. The division of ‘high-risk’ and ‘low-risk’ streams of care stretched the available staff and equipment:

*“Now [ ] WHO are saying that the COVID triage, the flow doesn't work properly, we need to change it all around, we need to put door exits at different places. [It's] a lot of work, more capital work. And a lot of work for the staff. Sometimes I question why we're separate [and] splitting our resources, splitting our staff, splitting our clinical equipment. Maybe we should be trying to manage it back at the ED. But that's up to [the Hospital Emergency Operations Centre]”*

Within PICT hospitals, increased attention on the ED also served to highlight challenges with overcrowding. In some cases, this resulted in hospital managers expressing interest in addressing longstanding challenges with access block (ie, delayed access to ward beds for admitted patients). Improved patient flow arrangements, such as expedited transfer of patients with confirmed COVID-19 to dedicated wards, was well received by EC clinicians:

*“So we had observers, and they talked about how the ED was overcrowded, and it's going to be worse with COVID, which I think is a good thing, because it's actually turning a spotlight on patient flow, which we needed anyway”*

Improvement in patient flow was not a universal experience, however. Despite the inherent transmission risks associated with ED crowding, some participants reported persisting access block. Across the region, a range of contributing factors were identified, such as a lack of ‘isolated’ beds on hospital wards; limited access to patient transport (especially in hospitals using off-site facilities to accommodate COVID-19 patients); and delays in laboratory results.

### Theme 3: developing standard operating procedures and checklists

PICT participants emphasised that a critical tool for effective COVID-19 response was the development and implementation of new standard operating procedures (SOPs) and management pathways. In Pacific settings with established EC systems, these could be adapted from existing resources:

*“We understand what we need to have to have an effective emergency care plan in place. So my role was replicating that emergency care plan but to COVID. So that's basically the approach that we took….we went from there into training in the facilities for clinical care, IPC [infection prevention and control], general safety of the staff, and then looking at restructuring the patient flow in facilities….How the patient come in, where they report to, the primary triage, and then from the primary triage, where do suspect cases get kept, do they get swabbed there? If they get swabbed and are mild, where do they go? Do they go home, or do they wait for the result? This was the work we did in all the facilities in the city.”*

In some health centres, checklists were developed to assist with critical processes, such as screening and streaming. These helped ensure consistency and safety:

*“We have a screening area in the main entrance to the emergency department. And we've been running training for the nurses that sit in front there too, and they have a checklist so that they could identify somebody [with] suspected COVID-19…We have a step-by-step list of things to do, who's going to do what, who's going to be called, who looks after this, the donning area for this place and where it would be”*

Many participants reported the value of exercises and ‘mock scenarios’ in implementing these new processes. Several low-resource settings described how they used in-situ simulation as a learning tool:

*“And these simulations, I'm very thankful that they were carried out. That helped us a lot. They provided insights for us in COVID triage [and] helped us to rearrange some of the flow that we had. Because when they presented with a patient who was unable to walk and had to be trolleyed in, we realised we needed to redefine some of our floor as well”*

In some settings, simulation exercises were inter-disciplinary and involved non-ED clinicians. These were useful in highlighting the intersection between EC and other health system components:

*“I'm not a part of the COVID-19 team... [but] I'm a part of the simulations. [In] the past few weeks, we've really looked at how the ED is going to respond, because we're the entrance to the hospital… So we've [done] quite a number of [ED] simulations. It's been going quite well”*

### Theme 4: maintaining ‘business as usual’

According to study participants, EC clinicians quickly realised that maintaining ‘business as usual’, as much as practicable, was critical to minimising the risk of unintended consequences. There was widespread concern that suspension of routine clinics, and the perceived ‘neglect’ of patients with chronic disease, was resulting in poor health outcomes. Several EC clinicians considered that decreased access to primary and secondary care had contributed to a rise in non-communicable disease exacerbation:

*“…They have stopped the routine surgeries, the routine clinics, and all, which has increased our burdens – so many patients who are coming with … intracranial haemorrhages, BKAs* [below knee amputations]*, the number of these critical patients have gone up because they don't have the access to the clinics to get their insulin, to get their antihypertensives”*

Additionally, there was concern that the overt focus on COVID-19 response was distracting clinicians and health administrators from supporting patients with other EC needs. Especially in PICT settings with limited resources and few COVID-19 cases, some participants were concerned that too much attention was being concentrated on the pandemic to the detriment of other priority areas:

*“Sometimes we were thinking ‘look it's good to focus on COVID’ but we have our common enemy we face every day, apart from that, we have TB. Malaria is almost eradicated in [our country], so TB is something we are still facing. And at the moment because it's rainy season, we also have dengue, and acute gastro; so it's very challenging, either you focus on COVID at the same time you have to focus on these day-to-day cases”*

Consistent with this, many clinicians also realised that processes designed to keep staff and patients safe were often accompanied by adverse effects. For instance, several participants reflected that screening at the point of hospital entry was impacting on access to routine healthcare:

*“Indirect effects for example are long lines because of pre-triage, our immunisation rates dropped for babies, because mothers bring their healthy babies to the lines, the lines are too long, they go back home; and they rather stay home. Our malnutrition rate at [the hospital] went from 11 to 24 percent for the same reason. They bring malnourished children in for review then don't come back because the lines are too long”*

Together, these realisations prompted clinicians to think deeply about the unintended consequences of the COVID response, and the need for proactive approaches to the assessment of ‘business as usual’ patients. This included redesigned processes for the reception and management of patients with urgent, non-COVID health conditions.

### Theme 5: Enhancing surveillance and data reporting

Finally, study participants reflected on data management challenges in resource-limited EC settings. They explained that, even under pre-pandemic routine operating conditions, there was limited data collection, analysis and reporting in many PICT EDs:

*“The person who was here before me was a [non-ED specialist]; imagine a [non-ED specialist] running the emergency department! There was not much data, I didn't even get a proper handover, and basically I started from zero. Now we have data because I always have a monthly report for our bosses… it's good that we are now up to date with our data”*

At the onset of the pandemic, many EDs were called upon to contribute to regional and national disease surveillance efforts. This included reporting of confirmed as well as suspected cases, using the principles of syndromic surveillance. For some participants, this catalysed data capture in the ED. It also highlighted the value of routine data collection, not just for surveillance purposes, but for improving performance:

*“Before COVID this department did have data but it's basic and not extensive like now. What we do now is use [our] data to now make recommendations.”*

Some participants were also called upon to integrate and report COVID-19 case data from surrounding and networked facilities. There were significant barriers to this process, mainly around communication and a lack of resources, especially in underdeveloped settings facing significant clinical service delivery pressures:

*“Reporting was quite a challenge, getting information from the 36 health facilities in the province and having to report for all of them to update the National Control Centre.”*

## Discussion

To our knowledge, this is the first in-depth, qualitative study to examine the application of ‘processes and data’ in PICT EC settings, and how they have been flexed and adapted during the COVID-19 pandemic. It has engaged diverse Pacific voices and drawn on the lived experience and unique perspectives of PICT EC clinicians and other key stakeholders.

From our analysis, five key thematic findings emerged that collectively reinforce the value of ‘processes and data’ in the delivery of safe and effective EC in the context of the pandemic. Specifically, implementing effective triage and screening (Theme 1), optimising patient flow (Theme 2), developing SOPs and checklists (Theme 3), maintaining ‘business as usual’ (Theme 4) and enhancing surveillance and data reporting (Theme 5) capacity represent areas of concern to PICT EC clinicians.

The study findings are broadly in keeping with the lessons learnt from other EC settings and regions during the pandemic.^12^, ^13^, ^14^^,^^23^^,^^36^, ^37^, ^38^ For instance, the value of dynamic strategies for triage and screening for COVID-19 was recognised early on by West African clinicians with recent experience of Ebola virus disease (EVD),^37^ and has been reinforced by experiences in North Africa.^36^ A recent consensus statement from a consortium of global critical care experts has also confirmed the role of simple triage instruments and clinical processes in responding to the pandemic.^14^ More specifically, the importance of tools that allow identification of patients at high risk of a positive diagnosis and/or clinical deterioration have been embraced by the global EC community.^39^^,^^40^

Recent analysis from the International Federation for Emergency Medicine has also highlighted the essential role of “infectious disease containment” strategies for EDs, including screening, streaming and zoning.^23^ The recognition of aerosols as the primary transmission vector for SARS-CoV-2 has reinforced the value of these approaches.^41^

Although these strategies have been widely adopted in high-income countries (HICs), the extent of their uptake in LMICs is unknown. As highlighted by this research, implementing screening, streaming and zoning in ED settings can be challenging, especially in the context of limited staff and poor infrastructure.^12^^,^^13^^,^^20^^,^^22^ For example, placing high transmission risk patients in negative pressure and/or individual rooms is a recommended strategy for HICs.^38^^,^^42^ However, this is not a practical solution for most resource-limited EDs, which rarely have individual cubicles (or equipment) and rely on open ward models to maximise the visibility of patients to clinical staff.^43^

To a large extent, the specific processes and data management solutions of concern to PICT clinicians during the pandemic reflect their pre-pandemic priorities. For instance, establishing effective systems for triage, patient flow and data collection are recognised priority areas for regional EC development.^15^^,^^44^ The pandemic has highlighted the intrinsic value of these processes, and the need for flexible approaches that can be adapted to evolving needs.^22^

Additionally, the realisation among PICT clinicians that public health interventions and EC process changes can impact on ‘business as usual’ is consistent with evidence from previous communicable disease outbreaks. Following the 2014-2016 EVD emergency in West Africa, for instance, indirect effects were thought to result in a higher burden of morbidity and mortality than the virus itself.^45^ These observations highlight the need for constant vigilance and dynamic approaches to EC and public health processes to mitigate the risk of unintended consequences.^12^

This theme also highlights the critical intersection between primary and emergency care.^2^^,^^46^ In many PICTs, access to community-based healthcare is limited, and patients often rely on EDs for ongoing care needs.^19^^,^^47^^,^^48^ In parallel, there is a high burden of non-communicable and chronic disease.^49^^,^^50^ It follows that public health measures that delay or compromise EC delivery for ‘business as usual’ patients are likely to impact on overall health outcomes.

Additionally, there is evidence that fear and anxiety related to COVID-19 can lead to delayed ED presentations for patients with acute illness.^51^ In settings where the ED fulfils both primary and secondary care needs, the impact of these changes in healthcare seeking behaviour is likely to be significant, and may seriously undermine attempts to ensure universal health coverage.^46^

The relatively small volume of discussion content related to ‘data’ is itself telling of the challenges for data collection in the ED. Although the value of EC registries has been clearly established, there are ongoing barriers to routine data collection in resource-limited EC settings.^16^^,^^52^ The pandemic has highlighted how systematised data management solutions can contribute to disease surveillance, but also help define broader epidemiological patterns and identify areas for performance improvement.

There was also a lack of participant discussion on how EC data and information processes, including morbidity and mortality reporting for COVID-19, were connecting to larger health information systems aimed at supporting evidence-based, national-level pandemic planning and response. Strong health information data collection and reporting arrangements, including the strengthening of civil registration and vital statistics systems, will be crucial to support PICTs in their ongoing multi-sectoral pandemic and emergency responses.^53^^,^^54^

There are several lessons that can be learnt from this research. These are summarised in Box 1**.** A key message is that processes and enablers that have been of value during the pandemic are likely to be impactful under normal conditions, and as PICTs emerge into the post-acute phase of the COVID-19 emergency. The corollary is that strengthening capacity for ‘routine’ EC is likely to result in more resilient systems, and enhance response capacity for future surge events, disasters and communicable disease outbreaks.^1^^,^^22^^,^^46^ This is particularly relevant in the Pacific context given the region's vulnerability to climate change and the associated risks for human health.^55^

Box 1 **Lessons learnt relevant to ‘processes and data’**

1. Processes and enablers that have been of value during the pandemic are likely to be impactful under ‘routine’ operating conditions, and contribute to safer and more efficient patient care.
2. Settings with pre-existing systems (for EC functions such as triage and data management) were better placed to ‘scale up’ their capacity and adapt to evolving needs.
3. Selected EC processes, especially triage and screening, were a high priority for EC clinicians responding to the pandemic, and are intrinsically linked to the ‘identity’ and function of the ED.
4. Whereas the clinical care of patients with COVID-19 is relatively straightforward (ie, the provision of oxygen and dexamethasone), implementing process changes in resource-limited settings can be complex and time-consuming, and relies heavily on strong EC leadership.
5. Data systems are under-developed in many PICT EDs, and there is huge potential to establish simple, low-cost data registries to enable surveillance and performance monitoring on an ongoing basis.

Alt-text: Unlabelled box

As discussed elsewhere, there are several limitations to this research.^17^ First, some PICTs were under-represented, and there was a predominance of Australian clinicians among the research team. This stands to bias the findings and limit the generalisability to certain Pacific LMICs. That said, our multi-disciplinary, cross-cultural research team reflexively sought to counter this by ensuring Pacific-based research team members were active partners at all stages of the research.

Second, the research was conducted during the first 18 months of the pandemic, and response priorities have evolved considerably over time. For instance, the COVID-19 Omicron variant is now predominant, and vaccination issues are of paramount concern to clinicians. At the time of data collection, these issues were not explored in detail, so further research, especially related to vaccine processes for staff and patients, is warranted.

Third, a majority of participants were hospital based, and pre-hospital EC ‘processes and data’ issues were underrepresented in informant discussion. This is reflected in an absence of data related to ambulance and inter-hospital retrieval processes.

Finally, while the building block framework has broad support across the region, it has been criticised for oversimplifying complex health systems, in which building block elements are interconnected and mutually dependent.^56^ As a result, the analysis in this paper may not reflect the multidimensional challenges that come with enhancing broad-based EC capacity.

This study is the first attempt to comprehensively document the experiences and priorities of Pacific EC providers responding to the pandemic. It provides evidence that a dynamic approach to EC processes is essential during communicable disease outbreaks, and that functional systems are critical to the delivery of safely and timely EC. Finally, it has highlighted the capacity of EDs to collect and report data that can contribute to disease surveillance, health service planning and performance improvement.

## Conclusion

The study has established that ‘processes’ and ‘data’ have been central to the delivery of safe and timely EC during the pandemic, just as they are under ‘routine’ operating conditions. Enabling EC clinicians to implement effective process and data management solutions will build resilience in EC systems.

## Contributors

RM, GOR, GP, CEB, SK and MC were primarily responsible for study design. DS, MK, PP and BK provided regional perspectives and contextual advice throughout all aspects of the project. All authors participated in data collection through online support forums, interviews and/or focus group discussions. LH was primarily responsible for transcription, preliminary coding and presentation of data to the broader research team. RM and GOR undertook the thematic analysis, secondary coding and synthesis for the ‘processes’ and ‘data’ building blocks, and LH, GP, DS, CEB, SK and MC contributed to data triangulation, amalgamation and integration. RM developed the first draft of this manuscript. The final version was reviewed and approved by all authors.

## Data sharing statement

Study protocols and de-identified data that underpin these findings may be available (between 9 and 24 months after publication) to investigators whose proposed use of these data has been approved by an independent review committee, subject to any restrictions imposed by relevant ethics committees, funders, the Australasian College for Emergency Medicine or the Pacific Community. Proposals to use the data may be submitted, and data made available, without investigator support.

## Declaration of interests

MC, GP, RM and GOR declare they are recipients of International Development Fund Grants from the Australasian College for Emergency Medicine Foundation. GP reports past research funding from the Pacific Community (SPC) and visiting Faculty status at the University of Papua New Guinea and Fiji National University. Additionally, RM reports grants from the Australian Government Department of Foreign Affairs and Trade as well as scholarships from the National Health and Medical Research Council (NHMRC) and Monash University. GOR reports that he is the recipient of a NHMRC Early Career Research Fellowship. CEB reports past research consultancy funding from SPC.
